# Validity of one‐time phantomless patient‐specific quality assurance in proton therapy with regard to the reproducibility of beam delivery

**DOI:** 10.1002/mp.17637

**Published:** 2025-01-27

**Authors:** Lukas Cornelius Wolter, Fabian Hennings, Jozef Bokor, Christian Richter, Kristin Stützer

**Affiliations:** ^1^ OncoRay – National Center for Radiation Research in Oncology Faculty of Medicine and University Hospital Carl Gustav Carus Technische Universität Dresden Helmholtz‐Zentrum Dresden – Rossendorf Dresden Germany; ^2^ Helmholtz‐Zentrum Dresden ‐ Rossendorf Institute of Radiation Oncology Dresden Germany; ^3^ Ion Beam Applications Particle Therapy GmbH c/o Universitätsklinikum Dresden Dresden Germany; ^4^ Department of Radiotherapy and Radiation Oncology Faculty of Medicine and University Hospital Carl Gustav Carus Technische Universität Dresden Dresden Germany; ^5^ German Cancer Consortium (DKTK) Partner Site Dresden and German Cancer Research Center (DKFZ) Heidelberg Germany

**Keywords:** Automation, Log file‐based dose reconstruction, Pencil beam scanning, Proton therapy, Quality assurance

## Abstract

**Background:**

Patient‐specific quality assurance (PSQA) is a crucial yet resource‐intensive task in proton therapy, requiring special equipment, expertise and additional beam time. Machine delivery log files contain information about energy, position and monitor units (MU) of all delivered spots, allowing a reconstruction of the applied dose. This raises the prospect of phantomless, log file‐based QA (LFQA) as an automated replacement of current phantom‐based solutions, provided that such an approach guarantees a comparable level of safety.

**Purpose:**

To retrieve a reliable LFQA conclusion from a one‐time plan delivery before treatment initiation, deviations between planned and logged parameters must either be persistent over all following treatment fractions or, in case of random fluctuations, must not have a relevant impact on the reconstructed dose distribution. We therefore investigated the reproducibility of log file parameters over multiple patient treatment fractions and compared the reconstructed dose distributions.

**Methods:**

Log file variability was examined at both spot parameter and integral dose levels. The log files of 14 patient treatment plans were analyzed retrospectively for a total of 339 delivered fractions. From the recorded *x*/*y* position and MU parameters per spot, the respective mean difference to the planned value (accuracy) and the standard deviation (reproducibility) were calculated for 108,610 planned spots. The dose distributions reconstructed from the log files of each fraction were evaluated against the planned fraction dose using 3D gamma index analysis. The dose‐based gamma pass rate Γ was correlated with a new spot‐based log file pass rate Λ. Beam timing information from the log files was used to quantify the total plan/field delivery time stability after excluding machine interlocks.

**Results:**

The mean spot‐wise accuracy with respect to distance from planned positions and MUs was (0.6 ± 0.3) mm and (0.0001 ± 0.0023) MU, respectively. The mean reproducibility of the observed single spot deviations was (0.2 ± 0.1) mm and (0.0004 ± 0.0004) MU (mean ± standard deviation). These variations resulted in minimal changes in the reconstructed fraction dose with Γ(2 mm/2%) > 99% for all studied fractions. Results for more sensitive criteria Γ(1 mm/1%) were plan‐specific, but on average > 92.6% per plan and correlated with Λ(1 mm) pass rates (0.51 ≤ *r*_Pearson_ ≤ 0.99). Field delivery times were reproducible within ± 4 s (2σ) and no treatment interruptions were observed in 92.8% of cases.

**Conclusions:**

The log file records of plan‐relevant spot parameters are well‐reproducible over multiple fractions and deviations have no dosimetrically relevant impact on the reconstructed fraction doses. Results of a one‐time pre‐treatment LFQA are considered as valid for the entire treatment course and there is no concern in this regard to replace state‐of‐the‐art phantom measurements in the current PSQA workflow.

## 1 INTRODUCTION

Pencil beam scanning (PBS) proton therapy (PT) enables highly‐conformal and effective treatments^1^ sparing healthy tissues and reducing side effects.^2^, ^3^, ^4^ Ensuring consistent high‐quality treatments requires rigorous quality assurance (QA), which currently involves machine‐specific measurements from daily to annual intervals^5^ and patient‐specific QA (PSQA), which is particularly vital for treatment approval.^6^ Traditional PSQA methods, relying on time‐consuming phantom‐based measurements,^7^ provide real dose measurements, but are limited to phantom materials and reduce available beamtime.

At the 2023 ESTRO Physics Workshop, consensus was reached to revise current PSQA workflows, aiming to replace manual procedures by automated, phantomless solutions like log file‐based QA (LFQA).^8^, ^9^, ^10^ LFQA utilizes treatment delivery log files from machine‐internal beam monitors, capturing parameters for each PBS spot, to perform dose reconstruction under actual treatment conditions (actual gantry angle and patient‐specific anatomical data) instead of performing phantom measurements at 0° gantry position.^11^ Although not providing absolute dose information, it offers great advantages over traditional methods for delivery performance assessment, such as enhanced error detection sensitivity compared to phantom measurement‐based PSQA.^12^

Log file information and subsequent QA procedures are subject to systematic and random uncertainties^13^ of the treatment delivery system itself and the beam monitors recording the data. Therefore, a one‐time pre‐treatment LFQA is only meaningful if the log file parameter values are reliable, that is, if deviations from planned values are systematically reproducible with minimal dosimetric impact of day‐to‐day fluctuations caused by machine performance and measurement uncertainties.

LFQA has been explored by several PT centers to analyze log file deviations and develop correction or prediction models.^14^, ^15^ It has already been implemented in multiple workflow scenarios in PT, reaching from full‐scale automated PSQA solutions^16^ over specialized applications such as 4D dose reconstruction for moving target irradiations^17^ to first online‐adaptive proton therapy (OAPT) workflows.^18^, ^19^ To the best of our knowledge, neither the reproducibility of single spot parameters over time nor resulting day‐to‐day changes in the reconstructed fraction dose have been published for actual patient treatment deliveries so far.

This study investigates the spatial and temporal variability of log file entries for repeated clinical PBS plan deliveries and characterizes deviations from planned values and their dosimetric impact using 3D gamma index analysis. The fraction‐wise gamma pass rates are correlated with a novel log file‐based passing criterion.

Symbolizing another step toward phantomless PSQA and improved understanding of the related uncertainties, this study further addresses a key aspect of future OAPT workflows, where the patient remains in treatment position during pre‐treatment PSQA. In this scenario, phantom measurements are impracticable and log file‐based QA becomes a valuable tool for post‐treatment treatment delivery monitoring.

## 2 METHODS

### 2.1 Treatment delivery system and log files

The IBA Proteus Plus (Ion Beam Applications, Louvain‐la‐Neuve, Belgium) PT system at the University Proton Therapy Dresden (UPTD) features a universal (PBS and double scattering‐capable) nozzle (for cGy/MU calibration details, see Supplement 1). It houses two identical strip‐segmented transmission ionization chambers (IC) for spot position measurements in the fast‐scanning *x*‐ and the slow‐scanning *y*‐direction (IEC‐61217 standard beam's eye view coordinate system, BEV). Lateral spot‐scanning positions are determined with micrometer resolution by fitting the charge distribution of the IC strips to a Gaussian curve.^20^ Treatment delivery log files contain beam parameters extracted from the ICs and data from various other sensors. One “record” log file containing plan‐relevant spot data at a sampling rate of 4 kHz is written for every energy layer, while one dedicated “events” log file is created for each delivered field and contains time‐resolved status and warning messages that allow for retrospective identification of treatment interruptions (interlocks).

### 2.2 Patient cohort and log file data extraction

Fourteen treatment plans for diverse tumor localizations were selected from patients treated with PBS PT at UPTD between 01/2020 and 01/2022. These plans were delivered for at least 15 fractions and featured various gantry angles, beam energies and spot numbers (Table 1). The dataset was complemented by the corresponding log files from a total of 339 delivered treatment fractions, resulting in 108,610 single spot statistics on (i) *x‐* and *y‐*position, (ii) monitor units (MU), and (iii) drill time. To compare the recorded spot positions with planned values, the average BEV (*x*, *y*)‐position per spot was extracted from the “record” file entries on IC level and projected into the isocenter plane using the intercept theorem^21^ (see Supplement 2). Spot MU was calculated from the charge accumulated in the integral IC plane during each spot delivery, using a machine‐specific conversion factor and conventional air density correction at delivery time. Beam timing and unintended interlocks were directly accessed via the timestamps of the log file entries and warning messages in the “events” log, respectively, excluding beam‐off time between field deliveries.

**TABLE 1:** PBS treatment plans sorted by body site (from cranial to caudal). Spot counts vary due to different target volumes, while the number of fractions equals the number of data points for the single spot statistics per plan.

ID	Tumor location	Gantry angles [°]	Number of spots	Number of fractions
1	Brain	200/290	3327	30
2	Brain	210/270/295	5758	30
3	Skull base	90/230/295	7114	25
4	Oral cavity	50/180/330	11135	15
5	Oral cavity	55/135/230/305	8521	25
6	Esophagus	135/210/225/335	5862	18
7	Esophagus	0/180	9314	15
8	Lung	100/340	4021	15
9	Lung	40/180/320	17843	32
10	Pancreas	144/219/280	6009	30
11	Pancreas	90/180/270	8030	30
12	Kidney	73/168/209	15772	25
13	Prostate	90/270	2961	29
14	Prostate	90/270	2943	20
Total		26	108610	339

### 2.3 Spot‐based log file analyses

The difference between the planned spot parameters $P=\{x_{\mathrm{plan}},y_{\mathrm{plan}},MU_{\mathrm{plan}}\}$ required for dose calculation and their log file values $L_{i}={\left\{x_{\log},y_{\log},MU_{\log}\right\}}_{i}$ in the *i* ^th^ delivered fraction (i=1…N) was calculated for every spot *s* as $L_{i,s}-P_{s}$. The accuracy of a parameter per spot was defined as the sample mean deviation from the planned value over all N≥15 fractions:

(1) $$\mu_{s}=\frac{1}{N}\sum_{i=1}^{N}L_{i,s}-P_{s}={\bar{L}}_{s}-P_{s}$$

The spot parameter reproducibility over all fractions was calculated as the corrected sample standard deviation of the *N* recorded values per spot:

(2) $$\sigma_{s}=\sqrt{\frac{1}{N-1}\sum_{i=1}^{N}{\left(L_{i,s}-{\bar{L}}_{s}\right)}^{2}}$$

Univariate analyses of the μ‐distributions at different gantry angles reveal systematic machine performance/beam monitoring deficiencies of the treatment delivery system. The respective σ‐distributions quantify the consistency of these deviations over all fractions, and hence, the reliability of arbitrary single spot deliveries. Both accuracy and reproducibility distributions were evaluated for the entire cohort, per plan and per gantry angle.

Additionally, the fraction‐wise fluctuation of total field delivery times was assessed, including the frequency of treatment interruptions due to machine interlocks.

### 2.4 Fraction‐wise pass rate analyses

Per treatment plan, *N* log file plans were created by overwriting the initially planned parameters for each spot with the log file information from the respective fraction. Dose reconstruction of these log file plans was performed on planning CT in the treatment planning system (Ray Station 11B‐R, RaySearch Laboratories, Stockholm, Sweden) using clinical Monte Carlo calculation settings and 3 mm dose voxel length. To quantify the clinical impact of log file deviations, reconstructed fraction doses were compared with the reference dose using 3D gamma index analysis.^22^, ^23^ Global 3D gamma pass rates (Γ) were calculated using 2%/2 mm and 1%/1 mm criteria, both with a low‐dose cutoff at 10% maximum dose and a voxel interpolation fraction of 10% of the distance to agreement (DTA). The reference dose maximum was chosen as a constant normalization value for all *N* fraction doses.

To study whether potential fraction‐wise fluctuations on dose level can also be detected using only log file data without patient‐specific information or dose re‐calculation, a spot‐based log file pass rate Λ(D) was defined as the MU ratio (% of the total fraction MU) of those spots delivered within a certain tolerance distance D to their reference position. Λ was evaluated for every delivered fraction *i* as:

(3) $$\Lambda_{i}\left(D\right)=\frac{\sum_{s\in S}MU_{i,s}\left[d_{i,s}<D\right]}{\sum_{s\in S}MU_{i,s}}$$

where *S* is the set of all recorded spots and $d_{i,s}=\sqrt{\Delta x_{i,s}^{2}+\Delta y_{i,s}^{2}}$ denotes the 2D Euclidian distance of the log file (*x, y*)‐position of spot *s* in the *i* ^th^ fraction to its planned position. Fraction‐wise log file pass rates were obtained with a tolerance distance of 1 mm [Λ (1 mm)] for each plan and were correlated with the respective Γ (1%/1 mm) pass rates using the Pearson coefficient $r_{\mathrm{Pearson}}$.

Both gamma and log file pass rates were calculated for all fraction‐wise log file plans, with the reference plan/dose being: (1) The original plan and dose distribution and (2) the reconstructed log file plan and dose distribution from the first fraction as a surrogate for a pre‐treatment LFQA delivery.

## 3 RESULTS

### 3.1 Spot position variation

**FIGURE 1:**
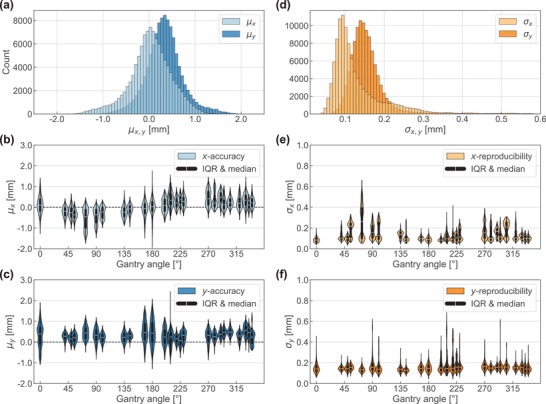
Single spot position accuracy ($\mu_{x}$, $\mu_{y}$; a) and reproducibility ($\sigma_{x}$, $\sigma_{y}$; d) extracted from the log files of all delivered fractions for all spots of the 14 treatment plans. Gantry angle dependence of *x* and *y* position accuracy (b, c) and reproducibility (e, f) distributions. White markers indicate the median, black areas indicate the inter‐quartile range (IQR) and black whiskers extend to furthest data point within 1.5 × IQR. Colored areas illustrate the shape of the underlying distribution. Outliers are not shown. Dashed lines indicate optimal agreement of log file parameters and planned values.

The spot‐wise position accuracies $\mu_{x}$ and $\mu_{y}$ over all fractions per plan showed symmetric distributions (Figure 1a) with mean/worst‐case values of 0.03 mm/−1.89 mm and 0.34 mm/2.44 mm in *x‐* and *y‐*direction, respectively (Table 2). Although the mean *y*‐records differed from the planned values, the standard deviations of the two accuracy distributions were similar for both coordinates (0.45 mm for *x*‐direction and 0.40 mm for *y*‐direction) and for 99.98% of spots the measured position was within 2 mm distance to the planned value.

**TABLE 2:** Mean and worst values for spot position accuracy and reproducibility in *x*‐ and *y*‐direction, Γ and Λ pass rates against the original plan as well as plan delivery time. The Pearson correlation coefficient $r_{\mathrm{Pearson}}$ of Γ(1%/1 mm) and Λ(1 mm) was evaluated for each plan individually.

ID	Mean (worst) position accuracy		Mean (worst) position reproducibility		Mean (min) Γ(1%/1 mm) [%]	Mean (min) Λ(1 mm) [%]	*r*_Pearson_	Mean (std. dev.) of delivery time
	$\mu_{x}$[mm]	$\mu_{y}$[mm]	$\sigma_{x}$[mm]	$\sigma_{y}$[mm]				*t* [s]
1	0.0 (−1.4)	0.5 (1.2)	0.1 (0.3)	0.2 (0.6)	98.2 (90.6)	73.2 (43.0)	0.78	70.45 (0.87)
2	0.3 (1.1)	0.3 (1.2)	0.1 (0.3)	0.2 (0.5)	97.6 (96.7)	84.6 (64.4)	0.77	111.34 (2.27)
3	0.1 (1.0)	0.4 (1.1)	0.1 (0.3)	0.2 (0.3)	99.0 (98.7)	93.5 (82.1)	0.51	118.59 (1.35)
4	0.1 (1.8)	0.4 (2.1)	0.1 (0.2)	0.1 (0.3)	94.3 (93.3)	76.1 (66.7)	0.77	186.72 (2.48)
5	0.0 (1.3)	0.3 (1.1)	0.2 (0.3)	0.2 (0.5)	98.1 (96.0)	93.8 (75.1)	0.97	226.06 (4.37)
6	0.1 (1.2)	0.2 (1.1)	0.1 (0.2)	0.1 (0.3)	99.0 (98.0)	95.8 (92.2)	0.63	138.15 (2.16)
7	0.1 (1.4)	0.4 (1.9)	0.1 (0.2)	0.1 (0.5)	92.6 (91.9)	71.0 (37.1)	0.56	127.95 (1.81)
8	0.0 (1.1)	0.2 (1.4)	0.1 (0.4)	0.2 (0.5)	95.2 (85.2)	88.1 (45.8)	0.99	120.11 (2.05)
9	−0.0 (−1.9)	0.3 (2.0)	0.1 (0.4)	0.2 (0.3)	95.4 (92.2)	80.7 (58.2)	0.93	218.85 (5.09)
10	0.1 (1.4)	0.2 (0.8)	0.1 (0.4)	0.2 (0.5)	99.4 (97.3)	83.7 (59.6)	0.83	103.6 (1.62)
11	0.1 (1.5)	0.3 (1.9)	0.2 (0.4)	0.2 (0.3)	97.5 (95.3)	68.7 (51.4)	0.80	144.15 (1.44)
12	−0.1 (−1.7)	0.4 (2.4)	0.2 (0.7)	0.2 (0.7)	97.6 (94.5)	63.6 (48.1)	0.91	200.39 (4.05)
13	−0.1 (−1.5)	0.1 (1.0)	0.2 (0.3)	0.2 (0.4)	93.6 (85.5)	54.6 (24.4)	0.88	54.82 (1.72)
14	−0.0 (−1.2)	0.5 (1.1)	0.2 (0.4)	0.2 (0.4)	94.9 (84.0)	57.1 (17.8)	0.88	64.03 (1.15)
All	0.0 (−1.9)	0.3 (2.4)	0.1 (0.7)	0.2 (0.7)	96.6 (84.0)	77.5 (17.8)	0.80	218.85 (5.09)

Γ … Gamma pass rate between planned and log file‐reconstructed fraction dose, Λ … Spot‐based log file pass rate.

The distributions of the spot‐wise reproducibility (Figure 1d) indicated day‐to‐day fluctuations of (0.13 ± 0.07) mm and (0.16 ± 0.06) mm (mean ± standard deviation) in *x*‐ and *y*‐direction, respectively (Table 2). That means both log file‐recorded spot position coordinates were equally well‐reproducible within the sub‐millimeter range over all delivered fractions (worst‐cases 0.66 mm and 0.68 mm in *x‐* and *y‐*direction, respectively).

The accuracy and reproducibility of log file positions were investigated as a function of the gantry angle to reveal machine‐specific systematic dependencies (Figures 1b, c, e, f). In *x*‐direction, spots delivered under horizontal gantry angles around 90° and 270° showed worse median spot‐wise position accuracies (Figure 1b) and also worse reproducibility (increased $\;\sigma_{x}$, Figure 1e). The more multimodal distributions under these angles indicated the existence of spot subsets with higher fluctuation upon repeated delivery. For *y*‐position accuracy (Figure 1c) and reproducibility (Figure 1f), no systematic angular dependence of the median values was observed, but the accuracy distribution widths (violin lengths) were higher for vertical gantry angles (around 0° and 180°).

### 3.2 Monitor units variation

With a mean accuracy $\mu_{\mathrm{MU}}$ of (−0.000 ± 0.002) MU (worst case: 0.047 MU), the expected difference between log file and planned parameters was only a fraction of the minimum deliverable spot MU of 0.014 MU. These offsets were precisely reproducible with a mean $\sigma_{\mathrm{MU}}$‐value of <0.001 MU. Following these observations, beam‐internal MU measurements are not expected to impact the reconstructed dose distribution. Also, the field‐wise MU accuracy and reproducibility distributions did not show angle‐specific effects.

### 3.3 Fraction‐wise pass rate analyses

Gamma analyses for all log file‐reconstructed dose distributions showed pass rates against the original plan of Γ(2%/2 mm) > 99.3% with minimal fluctuation across fractions. Day‐to‐day fluctuations in the reconstructed dose were revealed by Γ (1%/1 mm), with average passing rates > 92.6% being reproducible within at least ± 4.1% per treatment plan (Table 2, examplary log file‐reconstructed dose distribution in Supplement 3). The worst Γ(1%/1 mm) single fraction pass rate of 84.0% was obtained for a prostate plan (patient fourteen).

The spot‐based log file pass rates Λ (1 mm) against the original plan were on average > 54.6%, with high fraction‐wise fluctuations of up to ±21.2%. Importantly, plan‐wise Pearson correlation coefficients between Γ (1%/1 mm) and Λ(1 mm) of 0.51 ≤ $r_{\mathrm{Pearson}}$ ≤ 0.99 (mean 0.80) indicate strong correlation of the two different criteria.

When evaluated against the first delivered fraction as a surrogate for pre‐treatment LFQA deliveries, both pass rates returned (99.9 ± 0.5)% for all plans on average, with worst‐cases still scoring Γ (1%/1 mm) and Λ (1 mm) > 94.5%.

**FIGURE 2:**
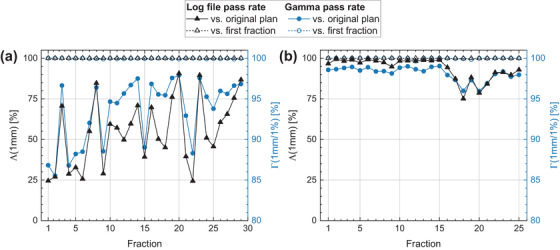
Fraction‐wise log file [black triangles, Λ(1 mm)] and gamma pass rates [blue circles, Γ(1%/1 mm)] with regard to the original plan (solid symbols) and the first delivered fraction (open symbols) for two selected patient plans. (a) Prostate, gantry angles [90°, 270°], patient‐ID 13. (b) Oral cavity, gantry angles [135°, 210°, 225°, 335°], patient‐ID 5. Reduced and strongly fluctuating pass rates are the result of impaired machine performance at horizontal gantry positions. A strong Pearson correlation was obtained between Λ and Γ against the original plan of $r_{\mathrm{Pearson}}$ = 0.88 (a) and $r_{\mathrm{Pearson}}$ = 0.97 (b). When comparing against the first delivered fraction, both criteria yielded pass rates > 99% for all fractions.

A prostate plan with two horizontal fields (Figure 2a) yielded a mean fraction pass rate against the original plan of Γ(1%/1 mm) = (93.6 ± 4.0)%, while a more complex oral cavity plan with four diagonal fields (Figure 2b) yielded Γ(1%/1 mm) = (98.1 ± 0.9)% on average. Lower mean pass rates and higher fluctuation across fractions confirm reduced spot position accuracy/reproducibility at horizontal gantry positions. Despite the inherent differences in underlying anatomy and field configuration, both cases show strong correlation between gamma and logfile pass rates against the original plan with Pearson coefficients of 0.88 and 0.97, respectively. One fraction each of patients seven and nine was aborted prematurely after 12/41 and 31/34 delivered PBS layers, respectively. These fractions were excluded from pass rate and beam timing analyses.

### 3.4 Beam timing

**FIGURE 3:**
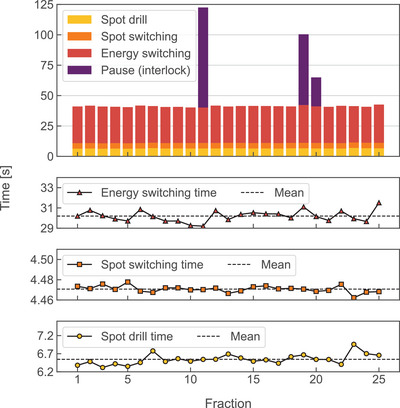
Fraction‐wise cumulative spot drill (yellow, circles), spot switching (orange, squares) and energy switching (red, triangles) times for one field with 27 energy layers (patient‐ID 3, gantry angle 230°). Beam interruptions due to machine interlocks (violet) were identified in fractions 11, 19, and 20. The total field delivery time excl. interlocks was (41.3 ± 1.28) s (mean ± double standard deviation).

The treatment delivery time, excl. interlocks and gantry rotation time, was reproducible within 2σ ≤ 3.64 s (single field) and 2σ ≤ 10.18 s (entire plan), indicating stable machine performance for the majority of investigated cases. An exemplary field of the skull base case (Figure 3) showed a total delivery time reproducibility of 0.64 s across all fractions, with the spot switching time being most precisely reproducible, followed by spot drill and energy switching time. Of all delivered fields, 92.8% did not show any treatment delays caused by machine interlocks. Patient seven exhibited the highest proportion of interlocks (11/15 fractions).

## 4 DISCUSSION

We systematically analyzed clinically delivered treatment plans from a heterogeneous patient cohort to quantify the accuracy and reproducibility of log file‐recorded spot positions, monitor units, and field delivery times. Unlike prior research, which primarily evaluated machine‐QA spot maps irradiated at single sessions, our work focuses on the reproducibility of patient treatment deliveries across the entire treatment course. Although there are fluctuations in the log file data, we demonstrated that a single pre‐treatment log file analysis reliably predicts the accuracy of all treatment fractions, a question that has not been addressed in previous studies. Additionally, we introduce the solely log file‐based pass rate Λ, a novel metric that shows patient‐specific correlation with the clinically‐established gamma pass rate. This enables rapid evaluation of delivery quality, even before the reconstructed dose is available, for example, by evaluating Λ(1 mm) against the first fraction or QA delivery. Lastly, our findings on beam timing demonstrate that 4D treatments do not require complex delivery timing approximation.

The predominant source of deviations in plan‐relevant log file parameters was the spot position recorded by nozzle‐internal ICs. Reconstructed fraction doses were evaluated with established dose‐based and novel log file‐based passing criteria to state the dosimetric impact of these effects. Notably, reconstructed fraction dose changes due to log file variability were detectable only with sensitive metrics [Γ(1%/1 mm), Λ(1 mm)], meaning the observed log file deviations have limited clinical relevance for the reconstructed dose.

The zero‐centered *x‐*position accuracy distributions result from canceling field‐specific gantry angle dependencies (negative and positive *x‐*position offsets for 90° and 270° gantry positions, respectively). The highest median shifts observed for horizontal fields are likely caused by a suboptimal gantry‐angle dependent beam transport through the gantry and potential additional mechanical effects, such as misalignment of the scanning magnets due to gravitational effects. Log file analyses indicated residual gantry angle‐specific spot position shifts. The detected effects are consistent with machine‐QA data, which also showed significantly increased *x‐*position deviations for gantry positions 90°/270° in comparison to 0° (see Supplement 4). Of note, with a recent update of the beam optics settings affecting the whole beam transport from cyclotron to nozzle, those effects have been reduced substantially.

Mean accuracy values of the *y‐*coordinate greater than zero can be associated with two phenomena (see Supplement 5): (1) Reproducible position offset of scanning rows in *y‐*direction due to scanning magnet calibration imperfections (systematic), and (2) degraded layer tuning prior to layer delivery causing a shift for all spots in the corresponding layer.

**FIGURE 4:**
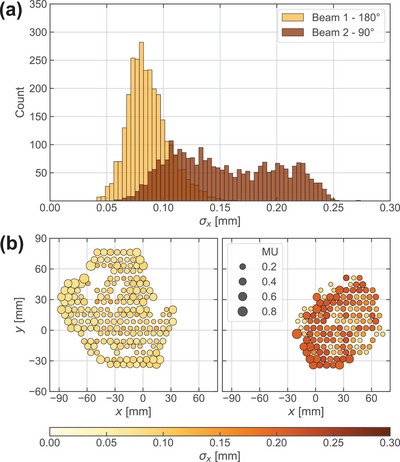
Spot *x‐*position reproducibility for two fields of an example pancreas plan (patient‐ID 11). $\sigma_{x}$ ‐distributions (a) for the vertical (180°, light yellow) and horizontal (90°, dark brown) fields of the same patient over all fractions. Spots of the horizontal field show worse reproducibility compared to the vertical field. Spot maps (b) of a selected layer with comparable beam energy reveal less reproducible high‐MU spots located at the lateral field edges of the horizontal field (90°, right) in comparison to the vertical field (180°, left).

Certain spot subsets exhibited stronger fraction‐wise fluctuations in their *x‐*coordinate (Figure 4a), particularly for horizontal gantry positions and high‐MU spots at the field edge (Figure 4b). This suggests less stable beam deflection for these spots, potentially due to longer delivery times of higher weighted spots. Overall, spot positions were precisely reproducible, suggesting that deviations from planned values are caused by systematically reduced delivery performance (e.g. for horizontal fields, high‐MU spots) rather than measurement uncertainties of the internal beam monitors. The IC‐recorded MU showed the best agreement with planned values, indicating high precision and reliability for detecting spot dose discrepancies in phantomless PSQA.

High 3D gamma pass rates were obtained for all delivered fractions, even with the sensitive 1%/1 mm criterion. With the 2%/2 mm criterion showing constant pass rates > 99% across fractions, the dosimetric influence posed by log file deviations may be deemed clinically insignificant.

The eligibility of the clinically established^24^ Γ(2%/2 mm)/Γ(1%/1 mm) for evaluation purposes within a future LFQA is questionable. These criteria introduce the DTA parameter to account for potential setup uncertainties, however, LFQA compares two calculated dose distributions on the same reference image, that is, without setup uncertainty. Therefore, a gamma criterion with reduced DTA (to account for remaining measurement uncertainties) or even voxel‐wise dose difference (DTA = 0) may be more suitable for LFQA.

Strong correlation of Λ(1 mm) and Γ(1%/1 mm) pass rates suggests that reconstructed dose differences can be directly attributed to beam position deviations on spot level. It has to be noted, that these correlations are plan‐specific, with Λ being anatomy‐independent while Γ depends on the planning image set used for dose calculation. Additionally, the fast‐accessible parameter Λ enables immediate feedback after each delivery, especially when being compared against clinically approved dose reconstructions from pre‐treatment PSQA deliveries. In that case, dose deviations would be detected by Λ(1 mm), without the necessity of fraction dose reconstruction or gamma index analysis.

Beam timing analysis revealed only minor day‐to‐day variations. This is of paramount relevance for treating anatomies that change with breathing as there is an interplay between the periodic target motion and the dynamic beam delivery.^17^ The interplay effect leads to unpredictable dose deteriorations because of variations in both breathing pattern and delivery timing. Showing that for the latter one the total fluctuations over the whole fraction delivery are much smaller than one breathing cycle, this study proves the appropriateness of using a fixed anticipated delivery time structure either during the optimization itself^25^, ^26^ or in prospective 4D robust dose evaluations.^27^, ^28^, ^29^ Instead of anticipating the delivery timing by a model of limited accuracy, it might be beneficial for some applications to even use the log file information from a single mock treatment.

Our findings align with previous studies of Toscano et al.^13^ where a log file spot position accuracy (mean difference to measurement) of 0.2 mm was obtained for an IBA Proteus Plus system. Dose‐volume‐histograms of the reconstructed and planned dose were compatible within the 95% confidence interval obtained from random log file uncertainties. Li et al.^8^ revealed gantry angle‐dependent systematic spot position offsets for a Hitachi PROBEAT system. In the same study, day‐to‐day fluctuations < 1 mm were recorded for both lateral spot position coordinates. Both conclude that the precision of beam‐internal ICs is sufficient for phantomless QA. A recent study by Ates et al.^30^ further confirmed neglectable differences between recorded and planned MU.

In summary, the present study demonstrates the appropriateness of a one‐time pre‐treatment phantomless PSQA based on treatment delivery log files, which is expected to enhance clinical workflows by optimizing the use of personnel, resources and beam availability, thereby eliminating the need for phantom‐based measurements. To maintain satisfactory log file results, we would recommend increasing the frequency of beam monitor QA procedures. Thanks to vendors facilitating access to treatment delivery log files for research and clinical purposes, LFQA methodologies can be advanced and more efficient PSQA workflows can be applied in the clinics. Beyond the enhancement of current QA workflows in conventional PT, this work provides key insights for future developments in OAPT, where log file‐based dose reconstruction is essential for automated PSQA due to the impossibility of phantom‐based measurements prior delivery.

## 5 CONCLUSIONS

The accuracy and reproducibility of treatment delivery log files from patient treatments have been systematically analyzed within a retrospective study. Discrepancies between log file entries and planned spot parameters were minimal and consistently reproducible. Day‐to‐day fluctuations had no significant dosimetric impact on reconstructed fraction doses, proving log files to be a reliable foundation for future phantomless PSQA implementations.

## CONFLICT OF INTEREST STATEMENT

OncoRay has a research agreement with and Jozef Bokor is an employee of Ion Beam Applications S/A. The authors have no conflicts of interest to disclose.

## Supporting information



Supplementary information

## Data Availability

Raw datasets used in this study are available from the corresponding author upon reasonable request.
