# Supplementary material for: Validity of one‐time phantomless patient‐specific quality assurance in proton therapy with regard to the reproducibility of beam delivery
Source: Med Phys. 2025 Jan 27;52(5):3173–82. doi: 10.1002/mp.17637 (PMC12059517; doi:10.1002/mp.17637)
Supplement: Supplementary file 1 — Supplementary information [file MP-52-3173-s001.pdf]

Supplement 1: Universal nozzle cGy/MU calibration

For the IBA universal nozzle commissioning at UPTD, cGy/MU conversion was determined by measuring the physical dose deposited by monoenergetic PBS reference fields of 10×10 cm^2 size (41×41 spot patterns with 0.25 cm quadratic spacing à 1 MU, totaling 1681 MU) at energy-specific depths in water with a PTW Semiflex chamber. The energy-dependent measurement positions were equal to [1cm + $R_{50}(E)$] / 2, yielding a physical dose of 0.108—0.112 cGy/MU in the plateau region of a pristine Bragg peak, with 0.9×10^8—1.6×10^8 ions/MU (Figure S1).

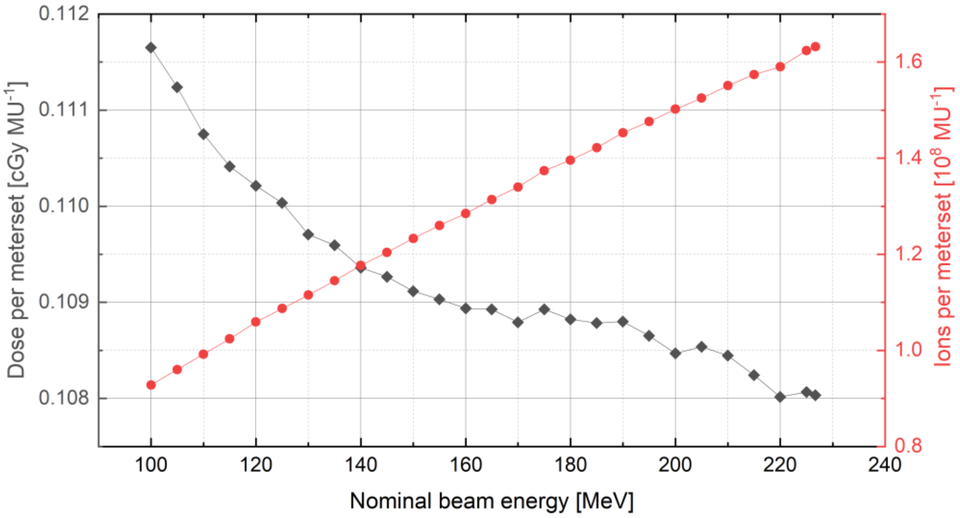

Figure S1: cGy/MU calibration curve of the IBA Dresden universal nozzle (PBS mode), measured at plateau level.

Supplement 2: Isocenter projection of lateral spot position

The spot position measurement in ionization chamber (IC) 2/3 is carried out horizontally (*x*-direction, fast scanning) by IC2 and vertically (*y*-direction, slow scanning) by IC3 in beam's eye view. This provides the spot position in the nozzle plane of the beam path (*z*-position of the ICs). The log file results must be converted into isocenter coordinates for evaluation. Using a theoretically defined, virtual source position in the beam path, this projection can be calculated using the intercept theorem (Figure S2).

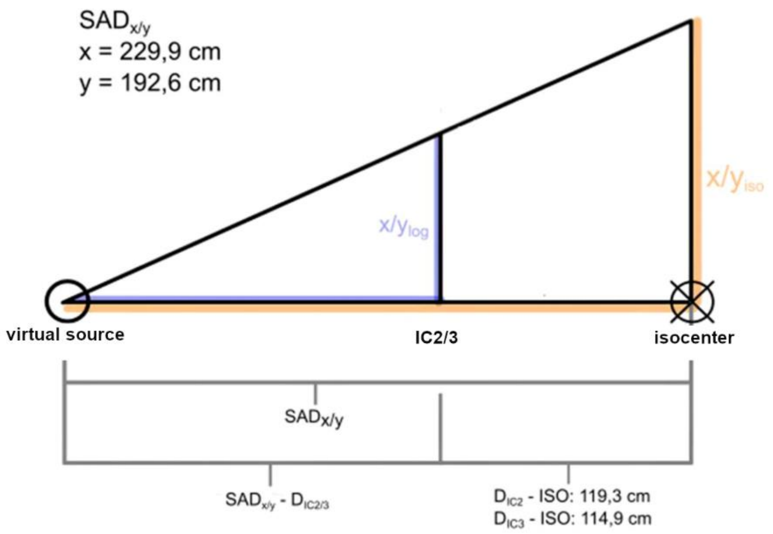

Figure S2: Projection of spot positions measured at nozzle level by IC2/3 (monitor chambers closest to the patient) to isocenter. The pencil beam originates from a virtual source position equal to the *z*-position of the deflected beam intersecting the central axis, resulting in different source-axis-distances (SADs) for projecting the *x*- and *y*-position of

the spot, due to different z-positions of the scanning magnets. The tuning-corrected spot position is scaled with the SAD and the isocenter distance of IC2/3 using the intercept theorem.

With respect to the tuning spot offset received on IC2/3, the spot position at isocenter depth can be calculated as:

$$x_{iso} = \left(x_{log} - \Delta x_{IC2}\right) \cdot \frac{SAD_x}{SAD_x - D_{IC2-iso}}$$

$$y_{iso} = \left(y_{log} - \Delta y_{IC3}\right) \cdot \frac{SAD_y}{SAD_y - D_{IC3-iso}}$$

Using:

- x_{log}, y_{log} … Lateral spot position obtained from the log file on IC2/3 level
- $\Delta x_{IC2}, \Delta y_{IC3}$ … Lateral spot map offset received during tuning on IC2/3 level
- $D_{IC2/3-iso}$ … z-distance between IC2/3 and isocenter

By using a constant virtual source position defined by the on-site team of the vendor, the necessity of evaluating IC1 feedback, as described in Lin et al (PMB 2016, 10.1088/1361-6560/aa5084), is bypassed. The quantities $X/Y_{\text{unscan, IC2/3}}$ in mentioned article are already included in the tuning pulse offsets $\Delta x/y_{\text{IC2/3}}$ in the above equations, obtained directly from log files.

Supplement 3: Log file dose difference distributions

The log file-reconstructed fraction dose of an example lung cancer treatment plan (patient-ID 8) yielded Γ(1%/1mm) and Λ(1mm) pass rates of 85.2% and 45.8%, respectively. Transversal 2D dose distributions (Figure S3) indicate over-/underdosage regions compared to the clinical plan, attributed to unexpectedly low spot position accuracy for said fraction/patient. Employing our clinical Gamma pass rate acceptance threshold of 95%, 4/15 fractions of that patient would have failed clinical PSQA, when comparing log file-reconstructed and initially planned dose.

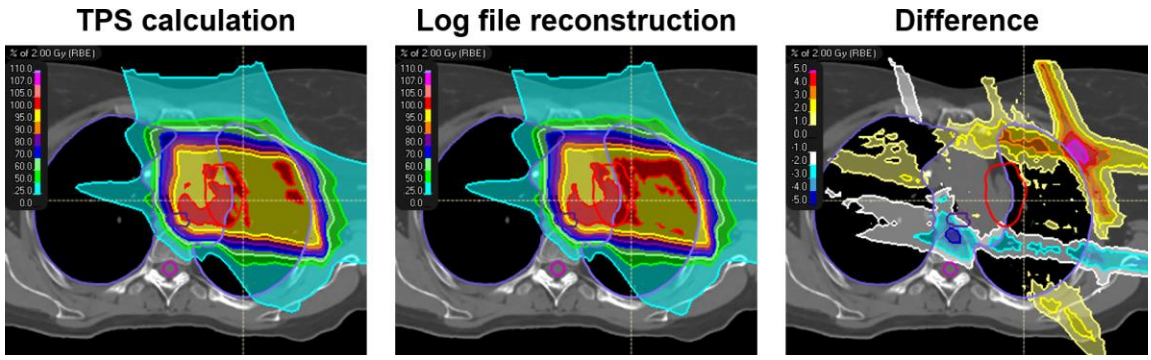

Figure S3: Fraction dose distributions of a lung cancer patient (patient-ID 8, fraction No. 12, transversal view of highest dose difference around the nodal CTV, cranial to the main CTV). Left: Planned dose. Center: Log file-reconstructed fraction dose. Right: Voxel-wise dose difference between log file-reconstructed and planned dose, showing overdosage of up to 6% of the maximum fraction dose of 2.0 Gy (0.12 Gy[RBE]).

Supplement 4: Log file results from machine-QA measurements

Another dataset originating from biannual machine-QA measurements (acquired from 2017-2023) was used for further investigations of the angular dependence of spot position deviations. Here, a standardized spot pattern consisting of 18 spots (Figure S4 left) was delivered under 8 gantry angles with an increment of 45° and measured with an IBA Lynx PT detector. From 829 of such spot maps (total 14922 spots), the spot positions obtained from the corresponding log files were compared to their planned values per gantry angle (Figure S4 right). Median spot position deviations in *x*-direction show sinusoidal dependence on the gantry angle.

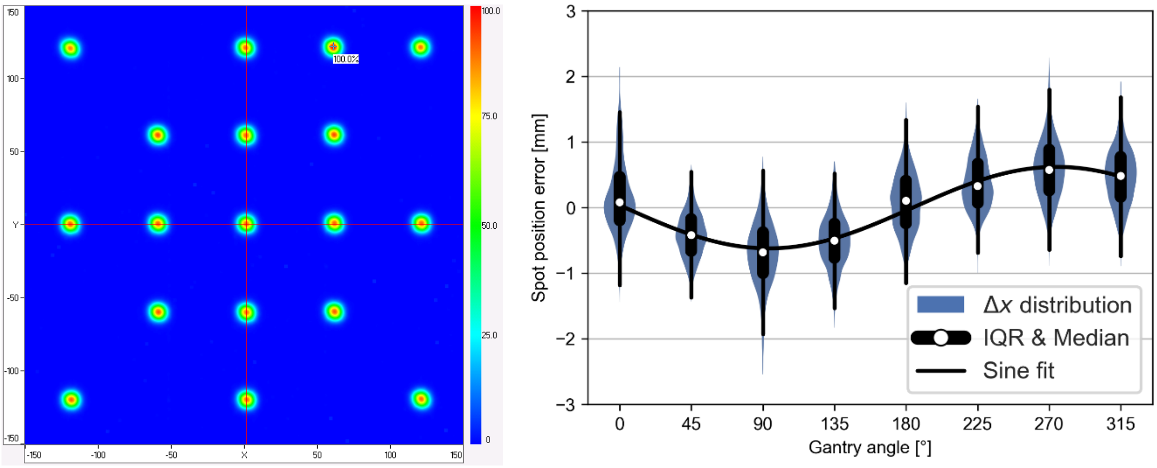

Figure S4: Left: Lynx-measured 2D intensity distribution of the machine-QA spot map, positions are obtained by local 2D gaussian fitting of the intensity profiles. Right: Distributions of *x*-position difference to planned values, per gantry angle. The differences between 90°/270° and 0° are significant ($p < 0.05$), supporting the angular dependence of the patient data in Section 3.1.

Supplement 5: Spot position shifts in *y*-direction

The *y*-direction direction represents the slow-scanning direction of the proton beam in IEC-61217 BEV coordinates, i.e. one *y*-magnet current is set for each “row” of spots, while the *x*-magnet (fast scanning direction) current switches continuously. Imperfections in scanning magnet calibrations therefore exert greater influence on the spot *y*-position, which is manifested by entire scanning rows showing a constant vertical position offset (Figure S5A). This effect is reproducible for every affected energy layer and hence deemed as systematic. On rare occasions, poor map tuning prior to field delivery may cause a random spot position offset of the entire affected energy layer, which is usually associated with repeated tuning attempts (Figure S5B).

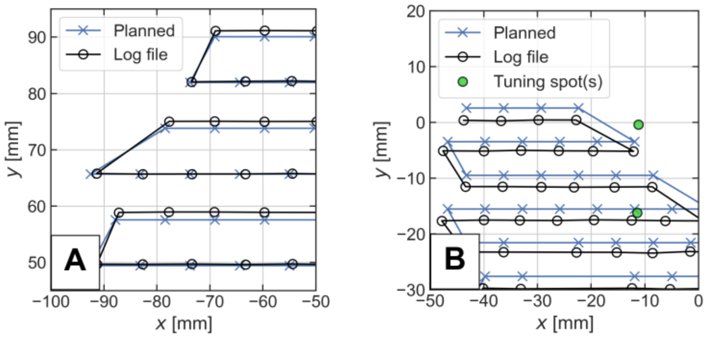

Figure S5: Log file spot positions (black) deviating from planned values (blue). A: Gantry angle 0°, beam energy 123.3 MeV. Spot row offsets in *y*-direction are a consequence of imperfections in *y*-scanning magnet calibration, representing a systematic, i.e. reproducible effect. B: Gantry angle 90°, beam energy 158.9 MeV. Isotropic spot map shift in *y*-direction as a consequence of randomly occurring tuning errors, indicated by multiple tuning spots.
